# X-ray PIV measurement of blood flow in deep vessels of a rat: An *in vivo* feasibility study

**DOI:** 10.1038/srep19194

**Published:** 2016-01-18

**Authors:** Hanwook Park, Eunseop Yeom, Sang Joon Lee

**Affiliations:** 1 Center for Biofluid and Biomimic Research, Department of Mechanical Engineering, Pohang University of Science and Technology (POSTECH), Pohang, 790-784, South Korea

## Abstract

X-ray PIV measurement is a noninvasive approach to measure opaque blood flows. However, it is not easy to measure real pulsatile blood flows in the blood vessels located at deep position of the body, because the surrounding tissues significantly attenuate the contrast of X-ray images. This study investigated the effect of surrounding tissues on X-ray beam attenuation by measuring the velocity fields of blood flows in deep vessels of a live rat. The decrease in image contrast was minimized by employing biocompatible CO_2_ microbubbles as tracer particles. The maximum measurable velocity of blood flows in the abdominal aorta of a rat model was found through comparative examination between the PIV measurement accuracy and the level of image contrast according to the input flow rate. Furthermore, the feasibility of using X-ray PIV to accurately measure *in vivo* blood flows was demonstrated by determining the velocity field of blood flows in the inferior vena cava of a rat. This study may serve as a reference in conducting *in vivo* X-ray PIV measurements of pulsatile blood flows in animal disease models and investigating hemodynamic characteristics and circulatory vascular diseases.

Circulatory vascular diseases such as atherosclerosis, stroke, and ischemic heart disease are the leading causes of death. Many factors are involved in the occurrence and progression of these circulatory diseases^1^. Among them, wall shear stress (WSS) is one of the important parameters affecting the occurrence of atherosclerosis^2^. WSS can be estimated from the velocity gradient near the vessel wall; thus, the velocity field of blood flows should be accurately measured under *in vivo* condition.

To obtain the velocity information of opaque blood flows in large blood vessel, many noninvasive imaging techniques such as X-ray PIV^3^, magnetic resonance imaging^4^,^5^, and ultrasound PIV^6^,^7^ have been introduced. High spatial resolution is essential to accurately assess WSS near the vessel wall. Thus, synchrotron X-ray PIV with high spatial resolution was developed^3^,^8^. This technique^8^,^9^ produces accurate measurement results on the velocity of blood flow in an artificial stenosed vessel model^10^ and *ex vivo* carotid artery^11^. However, fabrication of suitable tracer particles has been the most important issue in the *in vivo* measurement of blood flows using X-ray PIV technique. Thus, many biocompatible tracer particles, such as iopamidol encapsulated by polyvinyl alcohol^12^ and gold nanoparticles incorporated into chitosan^13^, have been introduced. These X-ray tracer particles are clearly distinguished by large absorption of X-ray beam. Tissues with high water content can induce significant X-ray beam attenuation. Therefore, tracer particles based on X-ray absorption are not suitable to measure blood flows under *in vivo* conditions due to X-ray attenuation by the surrounding tissues. To minimize such attenuation effects, CO_2_ microbubbles were fabricated as phase contrast based tracer particles^14^. Microbubbles are hollow, and their surface reflection provides high contrast and yields favorable measurement performance^15^. To investigate hemodynamic characteristics using X-ray PIV technique, ultrasound contrast agents of hollow type also have been used as tracer particles^11^,^16^. However, tracer particles used for X-ray PIV measurements have to be selected depending on experimental conditions and X-ray beam characteristics. In this study, therefore, CO_2_ microbubbles were used as flow-tracing particles. Nevertheless, the image contrast is decreased by the surrounding tissues because of the intrinsic features of X-ray beam. In our previous study^17^, using the X-ray PIV technique combined with CO_2_ microbubbles, we assessed the decrease of absorption contrast according to surrounding-tissue thickness. However, real blood vessels related with various cardiovascular diseases are usually located at deep positions in animal models. In addition, the absorption and phase contrasts of CO_2_ microbubbles in X-ray images are interrupted by the presence of tissues or organs which contain high water contents, including large attenuation in X-ray images. Therefore, X-ray PIV measurements of real blood flows in the deep blood vessels of animal models are required to assess the potential of this experimental technique for *in vivo* experiments.

The principal objectives of this study are to investigate the effects of surrounding structures on X-ray PIV measurements and determine the *in vivo* feasibility of using X-ray PIV to measure the velocity field of blood flow. First, the degree of X-ray image qualities was quantified by analyzing speckle contrast and size. The relationship between PIV measurement accuracy and X-ray image quality was explored, and the effects of surrounding structures were compensated by using additional image processing techniques. Subsequently, the velocity field of blood flow in the abdominal aorta of a rat cadaver was determined. The range of measurable velocity for the present X-ray PIV system with CO_2_ microbubbles as tracer particles was also determined in consideration of image contrast and measurement accuracy. This information is meaningful for the practical application of the x-ray PIV technique to animal models. Finally, we obtained the velocity information of blood flow in a live rat. These results would be helpful to investigate the hemodynamic characteristics of *rodent* atherosclerosis models *in vivo*.

## Result

### Image processing

**Figure 1:**
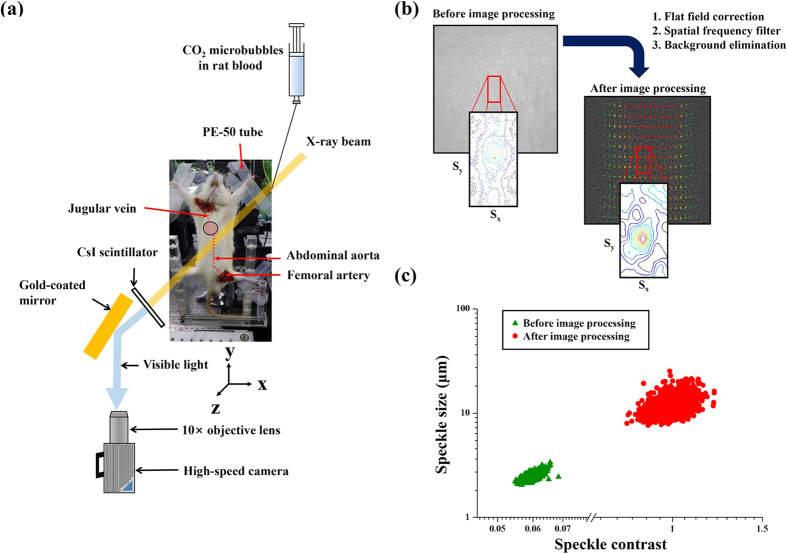
**(a)**Schematic of the experimental setup with a rat model. **(b)** Image prcessing procedure used in this study. Each contour plot shows a cross-correlation map of two consecutive images. **(c)** Comparison of speckle size and speckle contrast before and after image processing.

X-ray images of a blood flow in the abdominal aorta of a rat cadaver were obtained through synchrotron X-ray imaging. Figure 1a shows the schematic of the experimental setup used in this study. The blood seeded with CO_2_ microbubbles was supplied into the jugular vein of the rat cadaver by using a syringe pump. The working fluid passed through the heart and abdominal aorta, and then it was extracted from the femoral artery. The captured X-ray images contain information about the blood mixture and all surrounding structures along the pathway of X-ray propagation. Several digital image processing techniques were applied to compensate for the degradation of X-ray images caused by the surrounding structures. The effects of image processing on image qualities (speckle size and contrast) were quantified through speckle image analysis. The speckle size of CO_2_ microbubbles in X-ray images was evaluated using the normalized autocovariance function of speckle intensity distribution in the detection plane (x, y)^18^. The normalized autocovariance function 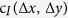 is described as follows:
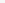



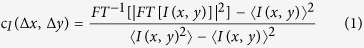


where FT indicates the Fourier transformation, I(x, y) is the intensity field of the image captured at the detection plane (x, y), and < > represents the ensemble averaging of images. Details about the procedure for speckle size evaluation can be found in our previous study^19^.

The image contrast of speckle patterns was also estimated through speckle image analysis with the followings equation:


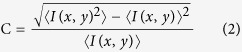


Figure 1b shows a typical image of CO_2_ microbubbles with corresponding velocity fields before and after image processing. A cross-correlation PIV algorithm was applied to two consecutive images to extract velocity information. In Fig. 1b, the cross-correlation maps of two consecutive interrogation windows for the raw and processed X-ray images are included. For the raw images, the peak position is unchanged from the center of the cross-correlation map because of low image contrast. This inaccurate cross-correlation analysis produces almost stationary velocity information, although flow motion is apparent in the consecutive flow images. Meanwhile, reasonable velocity information is successfully obtained from the processed X-ray images. Figure 1c represents the effect of image processing on the enhancement of speckle size and contrast. Image processing enhances both speckle size and contrast. Each data set was obtained from 100 X-ray images before and after image processing. Compared with the raw X-ray images, the processed X-ray images have significantly improved image quality.

### Velocity measurement in the abdominal aorta

**Figure 2:**
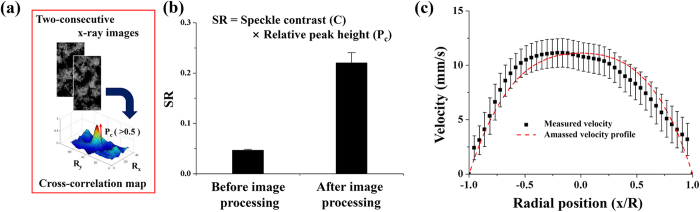
**(a)**Cross-correlation map with an explanation depicting the relative peak height. **(b)** SR value strongly depends on the image processing technique used. **(c)** Radial velocity profiles of blood flow in the abdominal aorta of a rat cadaver using CO_2_ microbubbles as tracer particles.

Figure 2a depicts a cross-correlation map with relative peak height (P_c_). P_c_ is closely related to the accuracy of X-ray PIV measurements. Figure 2b compares the SR values obtained before and after image processing. The SR value is defined as the product of P_c_ and speckle contrast (C). The SR value is largely increased after image processing. Figure 2c shows the centerline velocity profile measured at the abdominal aorta when the input flow rate is 0.3 mL/min. A fitting equation based on the bluntness index (*K*-value) was employed to estimate the real blood flow velocity based on the amassed velocity field derived from X-ray images. The red line indicates the amassed velocity profile fitted with a *K*-value of 3.31. The terminal velocity of CO_2_ microbubbles obtained by Stokes law^20^ was subtracted from the velocity field data to compensate the buoyance effect caused by CO_2_ microbubbles. This terminal velocity was estimated to be approximately 0.043 mm/s, which is less than 0.5% of the average velocity of the blood flow in the abdominal aorta.

### Maximum measurable velocity

**Figure 3:**
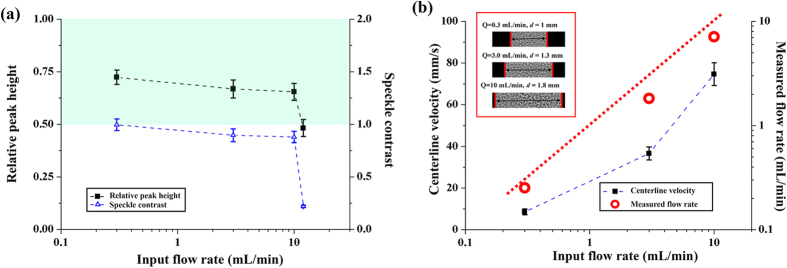
**(a)**Variations in relative peak height in cross-correlation map and speckle contrast of each interrogation window depend on input flow rate. Grean area indicates the region where P_c_ exceeds 0.5. **(b)** Variations in the centerline velocity and measured flow rate with respect to input flow rate. Dashed red line indicates the identical position between the input and measured flow rates.

The flow rate of blood injected by using a syringe pump (PHD 2000, Harvard Apparatus, USA) was varied (0.3, 3, 10, and 12 mL/min) to determine the maximum measurable velocity of blood flows in the rat model through the present experimental technique. The size of interrogation window was appropriately adjusted depending on the flow rate. The peak values in cross-correlation map (P_c_) and speckle contrast (C) were simultaneously considered as a function of the input flow rate to compare the measurement performance (Fig. 3a). P_c_ and C simultaneously decrease as the input flow rate increases. When the input flow rate becomes larger than 10 mL/min, the values of P_c_ and C are rapidly decreased. Based on our previous studies^17^,^21^, the measurement performance of X-ray PIV can be considered as reasonable, when P_c_ exceeds 0.5 (green area in Fig. 3a). For the velocity range having relatively high values of P_c_ (Q = 0.3, 3, 10 mL/min), the measured flow rate is compared with the input flow rate. Figure 3b shows the variations in centerline velocity (black quadrangle) and the measured flow rate (red circle) with input flow rate. The vessel diameter usually increases with input flow rate because of pressure increase in the blood vessels. The maximum measurable blood flow rate is approximately 7.1 mL/min, at which the centerline velocity is 74.64 mm/s, as determined using X-ray PIV with CO_2_ microbubbles in the rat model.

### *In vivo* feasibility test

**Figure 4:**
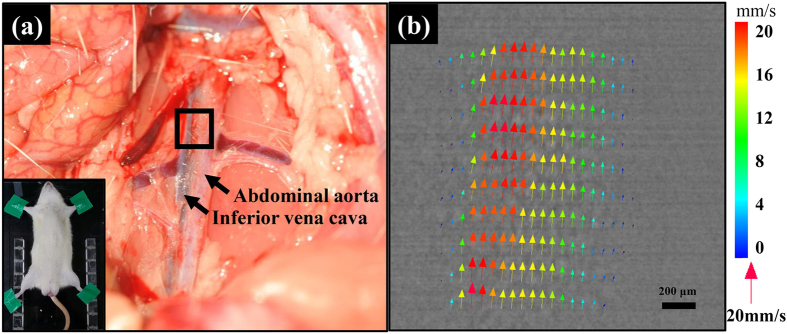
**(a)**Abdominal aorta and inferior vena cava in Sprague–Dawley rat (b)Instantaneous velocity field superimposed on the corresponding X-ray image of blood flow in the IVC of a live rat.

The velocity of blood flow under *in vivo* conditions within the range of measurable velocity can be determined using X-ray PIV. The velocity fields of blood flow were measured in the inferior vena cava (IVC) of the rat. Figure 4a shows abdominal aorta and IVC in Sprague–Dawley rat (SD rat). Figure 4b represents the measured velocity field of blood flow in the rat IVC. The mean peak velocity of the blood flow in the IVC of Sprague Dawley (SD) rat is about 100 mm/s^22^. Compared with the peak velocity of real physiological blood flow in the IVC, the maximum measurable velocity is approximately 75%. Therefore, we gave a surgical treatment to reduce blood flow velocity in the IVC. As shown in Fig. 4b, the measured velocity vectors are biased toward the left side because of the complicated vessel structures in the region near the IVC.

## Discussion

**Figure 5:**
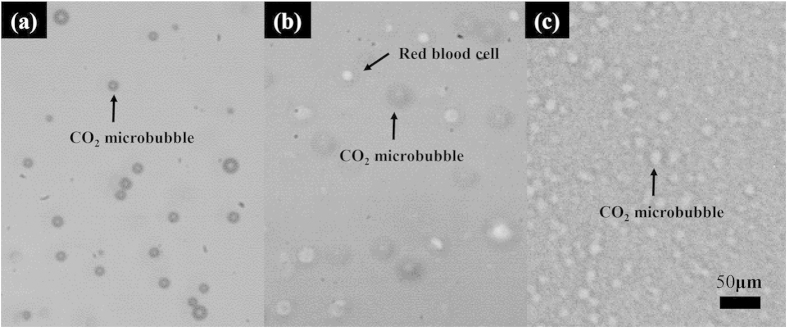
Optical image of (**a**) CO_2_ micorbubbles and (**b**) CO_2_ microbubbles with 2% HCT blood. (**c**) Typical X-ray image of CO_2_ microbubbles with 40% HCT blood.

Both absorption and phase contrasts contribute to X-ray imaging. Conventional X-ray imaging utilizes the attenuation of X-ray intensity caused by a test object located in the X-ray beam pathway. In synchrotron X-ray imaging experiments, a phase shift usually occurs when X-ray passes through an object. The complex refractive index (n) can be expressed as follows:


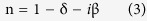


where *δ* indicates the decrement of the real part of the refractive index, and the imaginary part *β* describes the absorption index or extinction coefficient. In the hard X-ray region, the cross-section of phase shift (*δ*) for light elements is approximately 1000 times larger than that of absorption (*β*)^23^. Recent studies have developed CO_2_ microbubbles as phase contrast based flow tracers^14^. The visibility of CO_2_ microbubbles in blood is superior in X-ray images than in optical microscopic images (Fig. 5b,c). However, surrounding tissues around the blood vessels can significantly degrade the contrast of X-ray images. In the present study, we quantitatively analyzed the attenuation effects of surrounding tissues on the measurement performance of X-ray PIV. We also investigated the range of measurable velocity of blood flows in deep and complex blood vessels through a comparative examination between PIV measurement accuracy and speckle contrast. The signal-to-noise ratio (SNR) is commonly employed to quantitatively analyze image quality^24^. However, SNR is difficult to calculate in X-ray PIV experiments because particle images are required to have a Gaussian distribution and individual particles should be easily identified^25^. Neither of these requirements is satisfied by X-ray phase contrast images. The analysis of speckle dynamics is frequently used to study the motion of scattered objects or liquids. Thus, speckle contrast and speckle size instead of SNR were used in the present study to depict the effects of digital image processing.

Figure 1b,c demonstrate the effects of image processing. In PIV measurements, velocity vectors were obtained by applying a cross-correlation algorithm to two consecutive particle images. In general, the measurement accuracy of PIV is highly related to the quality of particle images^26^. When the PIV algorithm is applied to raw X-ray images, the cross-correlation peak is located in the vicinity of the central position in the cross-correlation map. This observation implies that speckle contrasts are not sufficient to identify the speckle patterns in X-ray images. This cross-correlation map indicates that the movement of tracer particles is almost stationary, although blood is continuously supplied into the rat cadaver through the syringe pump. Therefore, the measured velocity vectors are not well matched with the real displacement of tracer particles in the interrogation windows. Otherwise, the cross-correlation peak of the processed X-ray images is not located at the center of the cross-correlation map. The speckle patterns are recognized sufficient to determine the displacement of cross-correlation peak. The processed images may well indicate the displacements of tracer particles with significant accuracy. Therefore, speckle analysis strongly influences the measurement accuracy of PIV. Image processing also reduces the noises caused by image intensification to overcome insufficient X-ray beam flux. The effects of digital image processing on the measurement performance of X-ray PIV were investigated by measuring the whole velocity fields of blood flows in the abdominal aorta of the rat cadaver because surrounding tissues significantly attenuated the contrast of X-ray images. The measurement accuracy (SR) was estimated on the basis of the relative peak height (P_c_) in the normalized cross-correlation map and speckle contrast (C). P_c_ and C both strongly influence the measurement accuracy of PIV experiments. The measurement accuracy in the abdominal aorta is significantly enhanced after digital image processing (Fig. 2b). With the increase in SR, the velocity profiles of blood flows in the abdominal aorta can be correctly obtained using X-ray PIV with CO_2_ microbubbles. This result implies that image processing is essential for the *in vivo* X-ray PIV measurements of real blood flows. The measured velocity profile of blood flow in the abdominal aorta is asymmetric, which may have resulted from the nonsymmetric vessel configuration. The profile is not straight as the silicon tube (Fig. 4a) and has many branches. Although the velocity profile is not symmetric, *K*-value theorem was applied to obtain the average flow rate and the correction factor.

In speckle image velocimetry, the measurement accuracy is usually guaranteed when the P_c_ in the normalized cross-correlation map in two consecutive X-ray images exceeds 0.5 (green area in Fig. 3a)^27^. Therefore, the P_c_ for consecutive X-ray images was determined on the basis of flow rate to define the maximum measurable velocity range. Both P_c_ and C decrease with increasing input flow rate. In specific, P_c_ and C rapidly decrease when the input flow rate exceeds 10 mL/min. This observation implies that the measurement accuracy is largely decreased by the smearing effects on the X-ray images of a high-speed flow. Thus, the maximum measurable velocity that corresponds to the input flow rate of 10 mL/min should be determined. The flow rate estimated from the measured PIV results and vessel diameter was compared with the input flow rate to confirm whether or not the measurement performance of the present X-ray PIV technique is within the measurable velocity ranges. As shown in Fig. 3b, the vessel diameters increase with increasing flow rate. The compliance of blood vessels may contribute to the increase in vessel diameter in response to the change in hydrodynamic pressure. The measured flow rates are lower than the input flow rate for all cases tested in this study. The blood supplied to the rat cadaver model flows into several blood vessels, such as abdominal aorta, carotid artery, renal artery and others. Therefore, the flow rates measured in the abdominal aorta were below the input flow rate.

In this study, the maximum measurable velocity of blood flows in real blood vessels with surrounding tissues was investigated using X-ray PIV. We demonstrated the feasibility of the X-ray PIV technique combined with CO_2_ microbubbles to measure velocity field information of blood flows in the IVC of a live rat model with surgical intervention. Figure 4b shows a typical velocity field measured in the IVC of a live rat model. The velocity profile is shifted toward the left side of the IVC because the IVC is slightly curved (Fig. 4a). With a simple surgical treatment to reduce blood flow velocity in rat models, we could assess the feasibility of this X-ray PIV technique under *in vivo* conditions.

The temporal resolution^8^,^11^ of X-ray PIV measurements is affected by the experimental conditions such as X-ray beam flux, optical system arrangement and sensitivity of recording instruments. The maximum measurable velocity can be increased by enhancing these factors. For example, instead of monochromatic X-ray beam employed in this study, the use of polychromatic X-ray beam may increase X-ray beam flux significantly. Otherwise, the improvements of X-ray imaging instruments and digital image processing techniques also increase the range of measurable velocity^8^. Another crucial factors encountered in the application of this X-ray PIV technique for in *in vivo* measurements are dose limitation and heating effect of X-ray beam. These limitations would be overcome in the near future with technological advances in X-ray sources^28^ by which phase-contrast X-ray images can be acquired. Although the proposed technique still has somewhat technical limitations for investigating hemodynamic characteristic in a rat model under *in vivo* conditions, there limitations would be resolved in the near future with improving X-ray beam facilities.

In our previous study^13^, X-ray images of moving particles inside the cranial vena cava were consecutively captured and velocity vectors were obtained by dividing the length of each streaks by the exposure time. However, the shear stress acting on a vessel wall could not be evaluated because the number of velocity vector in the region near the vessel wall was rare and insufficient to get WSS. However, in the present study, we attempted to measure whole velocity field information of blood flows in a live rat model using X-ray PIV technique for the first time. Although some problems are still remained for the accurate estimation of shear rate and wall shear stress, such as the time-resolved detection of exact shape of a moving vessel wall and improvement of the algorithm for extracting amassed velocity profiles, the present X-ray PIV technique with CO_2_ microbubbles has a strong potential for *in vivo* measurements of real blood flows in animal models.

## Conclusion

This study demonstrated the feasibility of CO_2_ microbubbles streaming in a live rat model with real surrounding structures after applying digital image processing by conducting a comparative examination between speckle contrast and speckle size. The maximum flow velocity of the present X-ray PIV with CO_2_ microbubbles for measuring blood flows in deep vessels of a live rat model was approximately 76.64 mm/s. Finally, the feasibility of the present X-ray PIV technique for the *in vivo* measurement of real blood flows was demonstrated by measuring the velocity field information of blood flows in the IVC of a live rat model. The X-ray PIV technique combined with CO_2_ microbubbles can be used to measure velocity field information of blood flows in deep blood vessels, such as portal vein and renal vein in a rat. This study may serve as a reference in applying the present X-ray PIV system to animal disease models under *in vivo* condition and understanding the hemodynamic characteristics of circulatory vascular diseases.

## Methods

### X-ray imaging and image processing

X-ray PIV was performed at the 6C Biomedical imaging beamline of Pohang Light Source (PLS-II). The beam current and storage energy of the synchrotron facility are 360 mA and 3 GeV, respectively. The beam flux of a monochromatic X-ray beam is 1.2 × 10^12^ photon/s∙mm^2^. The median X-ray beam energy passing through a 1 mm thick silicon wafer is 23.8 keV. The size of the X-ray beam is 8 mm (H) × 5 mm (V). The sample was placed approximately 30 m downstream from the X-ray source. In general, the phase and absorption contrasts simultaneously appear in the captured X-ray images. The distance between the test sample and the scintillator is an important parameter for phase contrast X-ray imaging. This distance was fixed at 50 cm based on the result of a preliminary experiment. As the X-ray beam passes through a CsI scintillator of 500 μm in thickness, the X-ray beam is converted to visible light. X-ray images were consecutively acquired with a high-speed camera (SA 1.1, Photron, USA). The field of view is 1945 μm × 1945 μm (1024 × 1024 pixels) for 10× magnification. In this research, we used several image processing techniques, including a flat field correction(FFC), spatial frequency filtering, and background elimination. The FFC has widely been used in X-ray imaging experiments. It removes artifacts in 2D X-ray images, caused by inhomogeneous X-ray beam illumination. The background elimination method is usually employed to remove stationary structures in X-ray images. The mean background image was obtained by stacking 100 consecutive X-ray images. The number of stacking images was determined in consideration of structural movements of surrounding tissues and organs. Finally, the noises caused by the image intensifying process were largely reduced by adapting the spatial frequency filtering method^8^. The spatial frequency filtering technique employed in this study has been widely used in previous studies. The performance of this spatial filtering technique was assessed in detail in various PIV experiments such as conventional PIV^29^, X-ray PIV^8^ and echo-PIV^21^.

### CO_2_ microbubble generation

Mechanical agitation methods are one of the microbubble generation methods^30^,^31^. In this study, CO_2_ microbubbles were generated through mechanical agitation. About 5% human serum albumin and CO_2_ gas were mechanically agitated to generate CO_2_ microbubbles with a homogenizer (IKA-T25 digital ULTRA-TURRAX, IKA, Germany) operating at 15,000 rpm for 7 min. Details about the generation of microbubbles can be found in our previous study^14^. The average size of microbubbles used in this study is approximately 13.3 μm. Figure 5 shows the optical images of CO_2_ microbubbles (Fig. 5a), CO_2_ microbubbles in blood of 2% hematocrit (HCT) (Fig. 5b), and an X-ray image of 40% HCT blood (Fig. 5c).

### Experiment using rat cadaver

Figure 1a shows a schematic of the experimental setup involving a rat cadaver model. A male SD rat (12 weeks old, 373 g) was anesthetized with intramuscular injection of ketamine (100 mg/kg) and xylazine (10 mg/kg). A heparin-filled PE-50 tube (ID = 0.58, polyethylene tube) was cannulated into the right jugular vein. Approximately 500 IU/mL/kg heparin was injected into the right jugular vein to prevent blood coagulation. After 10 min injection of heparin, a PE-50 tube was cannulated into the femoral artery to extract blood from the rat model. The PE-50 tube cannulated into the jugular vein was connected with silicon tubes (ID = 1.5 and 0.8 mm). The mixture of CO_2_ microbubbles and rat blood was supplied into the rat model at various flow rates (0.3, 3, 10, and 12 mL/min) in a steady state by using a syringe pump (PHD 2000, Harvard Apparatus, USA) in order to compare the measured instantaneous velocity field with the input velocity information. The blood passed through the abdominal aorta and the blood was finally extracted from the femoral artery through a tube. All procedures performed on the animal models were approved by the Animal Care and Ethics Committee of POSTECH, and experiments were conducted in accordance with the approved guidelines.

### PIV measurement of amassed velocity profile

X-ray images were captured at a frame rate of 1000 frame per second (fps) for 7 s. Velocity field information was obtained by applying a two-frame cross-correlation PIV algorithm to the captured consecutive X-ray images. The size of the interrogation window for X-ray PIV measurements was determined in consideration of flow rate, speckle contrast, and relative peak height. It was 32 × 64 pixels with 50% overlapping when flow rate was 3 mL/min (Fig. 2a). The mean velocity field was obtained by ensemble averaging 200 instantaneous velocity fields. Various velocity components may be involved in the measured velocity information because each X-ray image contains all particles along the radial direction of X-ray propagation. A 2D amassed velocity field was obtained from the 3D volumetric flow information contained in the captured X-ray images. The amassed velocity profile for a Newtonian fluid^3^ was modified for blood flows^14^. In consideration of the shear thinning effect of real blood flows, the amassed velocity profile with bluntness parameter *K* can also be expressed using the following equation (Fig. 2b):


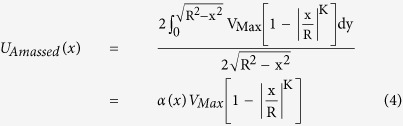


where *x* indicates the radial position. R and *K* are the radii of vessel and bluntness index, respectively. V_Max_ represents the maximum velocity of blood flow. Simulation was performed to acquire α at specific *r* and *K* because the correction factor (α) was dependent on the radial position and *K*. Details about the amassed velocity profile can be found in our previous study^14^.

### *In vivo* experiment using a rat model

To minimize the cardiac load induced by surgical intervention, the IVC located in the deepest position of rat model was selected to demonstrate the feasibility of this X-ray PIV measurements technique under *in vivo* conditions. Similar to the preparation of a rat cadaver, a male SD rat (12 weeks old, 395 g) was anesthetized, and heparin (500 IU/mL/kg) was injected into the right femoral vein to prevent blood coagulation. At 10 min after heparin injection, the left femoral vein was tied with a thread to decrease blood flow velocity in the IVC. The PE-50 tube cannulated in the right femoral vein was connected with silicon tubes (ID = 1.5 and 0.8 mm) to supply the mixture of CO_2_ microbubbles and rat blood into the rat by using a syringe pump (PHD 2000, Harvard Apparatus, USA). The injection flow rate was fixed at 0.1 mL/min. All procedures performed on animal models were approved by the Animal Care and Ethics Committee of POSTECH, and the experiments were carried out in accordance with the approved guidelines.

## Additional Information

**How to cite this article**: Park, H. *et al*. X-ray PIV measurement of blood flow in deep vessels of a rat: An *in vivo* feasibility study. *Sci. Rep.* **6**, 19194; doi: 10.1038/srep19194 (2016).

## References

[b1] Cecchi E. . Role of hemodynamic shear stress in cardiovascular disease. Atherosclerosis 214, 249–256 (2011). 20970139 10.1016/j.atherosclerosis.2010.09.008

[b2] Malek A. M., Alper S. L. & Izumo S. Hemodynamic shear stress and its role in atherosclerosis. J. Am. Med. Assoc . 282, 2035–2042 (1999). 10.1001/jama.282.21.2035 10591386

[b3] Lee S. J. & Kim G. B. X-ray particle image velocimetry for measuring quantitative flow information inside opaque objects. J. Appl. Phys. 94, 3620 (2003).

[b4] Markl M. . *In vivo* wall shear stress distribution in the carotid artery effect of bifurcation geometry, internal carotid artery stenosis, and recanalization therapy. Circ. Cardiovasc. Imaging 3, 647–655 (2010). 20847189 10.1161/CIRCIMAGING.110.958504

[b5] Lorenz R. . 4D flow magnetic resonance imaging in bicuspid aortic valve disease demonstrates altered distribution of aortic blood flow helicity. Magn. Reson. Med. 71, 1542–1553 (2014). 23716466 10.1002/mrm.24802 PMC3778148

[b6] Gharib M. & Beizaie M. Correlation between negative near-wall shear stress in human aorta and various stages of congestive heart failure. Ann. Biomed. Eng. 31, 678–685 (2003). 12797617 10.1114/1.1574025

[b7] Kim H. B., Hertzberg J., Lanning C. & Shandas R. Noninvasive measurement of steady and pulsating velocity profiles and shear rates in arteries using echo PIV: *in vitro* validation studies. Ann. Biomed. Eng. 32, 1067–1076 (2004). 15446503 10.1114/b:abme.0000036643.45452.6d

[b8] Jung S. Y., Park H. W., Kim B. H. & Lee S. J. Time-resolved X-ray PIV technique for diagnosing opaque biofluid flow with insufficient X-ray fluxes. J Synchrotron Radiat 20, 498–503 (2013). 23592630 10.1107/S0909049513001933

[b9] Lee S. J. & Kim G. B. Synchrotron microimaging technique for measuring the velocity fields of real blood flows. J. Appl. Phys. 97, 064701 (2005).

[b10] Jamison R. A., Dubsky S., Siu K. K., Hourigan K. & Fouras A. X-ray velocimetry and haemodynamic forces within a stenosed femoral model at physiological flow rates. Ann. Biomed. Eng. 39, 1643–1653 (2011). 21293973 10.1007/s10439-011-0260-2

[b11] Jamison R., Siu K., Dubsky S., Armitage J. & Fouras A. X-ray velocimetry within the ex vivo carotid artery. J. Synchrotron Radiat. 19, 1050–1055 (2012). 23093769 10.1107/S0909049512033912

[b12] Lee S. J., Jung S. Y. & Ahn S. Flow tracing microparticle sensors designed for enhanced X-ray contrast. Biosens. Bioelectron . 25, 1571–1578 (2010). 20022479 10.1016/j.bios.2009.11.010

[b13] Jung S. Y., Ahn S., Nam K. H., Lee J. P. & Lee S. J. *In vivo* measurements of blood flow in a rat using X-ray imaging technique. Int. J. Cardiovasc. Imaging 28, 1853–1858 (2012). 22354531 10.1007/s10554-012-0029-1

[b14] Lee S. J., Park H. W. & Jung S. Y. Usage of CO_2_ microbubbles as flow-tracing contrast media in X-ray dynamic imaging of blood flows. J. Synchrotron Radiat. 21, 1160–1166 (2014). 25178007 10.1107/S1600577514013423

[b15] Kim G. B., Lim N. Y. & Lee S. J. Hollow microcapsules for sensing micro-scale flow motion in X-ray imaging method. Microfluid. Nanofluid. 6, 419–424 (2009).

[b16] Dubsky S. . Computed tomographic x-ray velocimetry. Appl. Phys. Lett. 96, 023702 (2010).

[b17] Park H., Yeom E., Seo S. J., Lim J. H. & Lee S. J. Measurement of real pulsatile blood flow using X-ray PIV technique with CO_2_ microbubbles. Sci. Rep . 5, 8840 (2015). 25744850 10.1038/srep08840 PMC4351547

[b18] Piederrière Y. . Scattering through fluids: speckle size measurement and Monte Carlo simulations close to and into the multiple scattering. Opt. Express 12, 176–188 (2004). 19471524 10.1364/opex.12.000176

[b19] Yeom E. & Lee S. J. Microfluidic-based speckle analysis for sensitive measurement of erythrocyte aggregation: A comparison of four methods for detection of elevated erythrocyte aggregation in diabetic rat blood. Biomicrofluidics 9 (2015). 10.1063/1.4917023 PMC4385097 25945136

[b20] Lee S. J. & Kim S. Simultaneous measurement of size and velocity of microbubbles moving in an opaque tube using an X-ray particle tracking velocimetry technique. Exp. Fluids 39, 492–497 (2005).

[b21] Yeom E., Nam K. H., Paeng D. G. & Lee S. J. Improvement of ultrasound speckle image velocimetry using image enhancement techniques. Ultrasonics 54, 205–216 (2014). 23725769 10.1016/j.ultras.2013.05.001

[b22] Chang C.-J. . Differential endothelial gap junction expression in venous vessels exposed to different hemodynamics. J. Histochem. Cytochem. 58, 1083–1092 (2010). 20805582 10.1369/jhc.2010.956425 PMC2989245

[b23] Momose A. & Fukuda J. Phase‐contrast radiographs of nonstained rat cerebellar specimen. Med. Phys. 22, 375–379 (1995). 7609717 10.1118/1.597472

[b24] Meinhart C., Wereley S. & Gray M. Volume illumination for two-dimensional particle image velocimetry. Meas. Sci. Technol. 11, 809 (2000).

[b25] Antoine E. . Flow measurements in a mlood-perfused collagen vessel using x-ray micro-particle image velocimetry. PLoS One 8, e81198 (2013). 24260559 10.1371/journal.pone.0081198 PMC3832459

[b26] Hart D. P. PIV error correction. Exp. Fluids 29, 13–22 (2000).

[b27] Yeom E., Nam K. H., Paeng D. G. & Lee S. J. Effects of red blood cell aggregates dissociation on the estimation of ultrasound speckle image velocimetry. Ultrasonics 54, 1480–1487 (2014). 24794508 10.1016/j.ultras.2014.04.017

[b28] Lundström U. . X-ray phase-contrast CO2 angiography for sub-10 μm vessel imaging. PMB 57, 7431 (2012). 10.1088/0031-9155/57/22/7431 23093393

[b29] Shavit U., Lowe R. J. & Steinbuck J. V. Intensity Capping: a simple method to improve cross-correlation PIV results. Exp. Fluids 42, 225–240 (2007).

[b30] Xu Q., Nakajima M., Ichikawa S., Nakamura N. & Shiina T. A comparative study of microbubble generation by mechanical agitation and sonication. Innov. Food Sci. Emerg. Technol. 9, 489–494 (2008).

[b31] Jeon D. S. . The usefulness of a 10% air-10% blood-80% saline mixture for contrast echocardiography: Doppler measurement of pulmonary artery systolic pressure. J. Am. Coll. Cardiol. 39, 124–129 (2002). 11755297 10.1016/s0735-1097(01)01698-9

